# The Effect of Environmental Regulation on Employment in Resource-Based Areas of China—An Empirical Research Based on the Mediating Effect Model

**DOI:** 10.3390/ijerph14121598

**Published:** 2017-12-19

**Authors:** Wenbin Cao, Hui Wang, Huihui Ying

**Affiliations:** Department of Management Science and Engineering, School of Business, Jiangnan University, 1800 Lihu Avenue, Wuxi 214122, China; wanghui20171114@163.com (H.W.); 18352537943@163.com (H.Y.)

**Keywords:** employment effect, environmental regulation, mediating effect, system GMM

## Abstract

While environmental pollution is becoming more and more serious, many countries are adopting policies to control pollution. At the same time, the environmental regulation will inevitably affect economic and social development, especially employment growth. The environmental regulation will not only affect the scale of employment directly, but it will also have indirect effects by stimulating upgrades in the industrial structure and in technological innovation. This paper examines the impact of environmental regulation on employment, using a mediating model based on the data from five typical resource-based provinces in China from 2000 to 2015. The estimation is performed based on the system GMM (Generalized Method of Moments) estimator. The results show that the implementation of environmental regulation in resource-based areas has both a direct effect and a mediating effect on employment. These findings provide policy implications for these resource-based areas to promote the coordinating development between the environment and employment.

## 1. Introduction

It has become abundantly clear that fossil fuel powered industrialization has had unanticipated adverse environmental impacts. One of the most significant challenges faced by global leaders today is how to achieve inclusive and sustainable industrial development, thereby creating jobs and reducing poverty, while combating climate change and resource depletion. Many industrialized countries have developed a series of environmental regulation policies to promote the coordinated development of employment and environment. For example, confronted with excessive consumption of resources and environmental damage caused by the manufacturing industry, the Chinese government has enacted a series of laws and regulations to protect environment such as the Administrative Regulation on Levy and Use of Pollutant Discharge Fee (2003), the Law on the Prevention and Control of Environmental Pollution by Solid Waste (2004), the Law on Conserving Energy (2007), the Measures for the Disclosure of Environmental Information (for trial implementation) (2007), the Circular Economy Promotion Law (2008), the Measures for Environmental Administrative Punishment (2010), the Atmospheric Pollution Prevention and Control (2015 Revision), etc., since 2003. Meanwhile, the debate on whether the implementation of an environmental regulation policy will downsize the scale of employment has never stopped. In fact, the economic development in China has entered a new norm since 2012. The mode of economic growth began to change and the growth of GDP (Gross Domestic Product) gradually slowed down. However, the employment rate has not slowed down along with GDP. Since 2013, the number of new jobs in urban areas has reached about 13 million people per year. In 2014, China made the goal of creating more than 10 million new jobs and has introduced many employment promotion policies. The government is trying to adjust the industrial structure and create more jobs to adapt to the new situation of economic development. According to the National Bureau of Statistics, the employment rate has increased rapidly in the service sector since 2012. Over the past few years, the employment growth rate in the service sector has far exceeded the rate in the manufacturing industry. However, the trend of employment growth in most resource-based areas is not obvious. Our research aims to explain whether the environment regulations affect employment, especially in resource-based areas of China.

Environmental regulation affects employment by increasing the cost of production and by directly promoting the development of an environmental-protection industry. Towards the direct employment effects of environmental regulation, Rolf found that the firms under strict environmental regulations had a higher tendency to increase employment in Norway [1]. Greenstone estimated the impacts of the Clean Air Act based on 1.75 million plant observations from the Census of Manufactures, and found that related areas lost approximately 590,000 jobs [2]. Curtis used the Three-Differences model to examine the effect of the policies on the nitrogen oxide emissions trading on labor demand in the United States from 1998 to 2009, and found that the nitrogen oxide emissions trading made the employment rate of the high-energy-intensity manufacture industry decrease by 3.9% [3]. Based on the CGE (Computable General Equilibrium) method, Dissou found that the effect of reducing carbon emissions on employment is negative [4]. Gray applied the Difference-in-Differences estimator to estimate the causal effect of the Cluster Rule on employment in the pulp and paper industry, and found that the policies had relatively small positive effects on the employment rate [5]. Ira Altman analyzed the projects of capturing carbon to implement low-carbon power generation and verified that the positive effect on employment is significant [6]. Horbach found that the effects of environmental regulation on employment are related to the type of technological innovation. If the technological innovation takes place during the production process, it has a positive impact on employment, while if the technological innovation takes place in the end treatment, the impact on employment is negative [7]. Morgenstern’s analysis shows that increasing environmental spending does not have a significant impact on employment. With a net increase of 1.5 jobs per 100 million dollars, the effect is slight [8].

While the direct effects of environmental regulation on employment have been widely studied in recent years, studies on mediating the effect that environmental regulation has on employment are rare. Porter argued that the regulated enterprises must transform and upgrade their equipment and technology, and increase the energy efficiency. Meanwhile, environmental regulation can stimulate enterprises to optimize their resource allocation, improve management efficiency, and reduce inefficient factors in the production process. Environmental regulation can not only improve environmental quality, but can also promote industrial development and economic growth [9]. Fullerton believed that the impact of environmental regulation on industrial structure depends on the behavior of enterprises. As the result of environmental regulation, the price of the production factor rises, and it leads to the adjustment of production behavior [10]. Zhonghua Cheng argued that the substitution effect of environmental regulation has promoted the upgrading of the industrial structure. The manufacturing industry is more likely to be affected by environmental regulation than the service industry. From the perspective of the theory of consumer choice, more services will be chosen. The substitution is conducive to the development of the service industry, and promotes the upgrading of the industrial structure [11]. Jaffe and Palmer found that environmental regulation could stimulate enterprises to carry out technical innovation, and the relationship between environmental regulation and industrial R & D is significantly positive. They also pointed out that the effect of environmental regulation on technical innovation is the “weak” version of the Porter hypothesis [12]. The recent research of the United Nations Industrial Development Organization (UNIDO) and the Global Green Growth Institute (GGGI) shows that there are clear net gains in employment generation, in shifting from conventional energy sources to renewable energy sources [13]. Zhang Juan established the threshold model based on the 33 resource-based cities’ data. He found the nonlinear relationship between environmental regulation and employment, where only when the proportion of the third industry and the industrial profit margin exceeds a certain level, will the environmental regulation have a positive impact on employment [14]. All the papers mentioned above used industrial structure or technological innovation as the control variable. It is not enough to examine the mediating employment effects based on the two variables. This paper argues that environmental regulation can have an impact upon employment by promoting the upgrade of the industrial structure and technological innovation.

**Figure 1:**
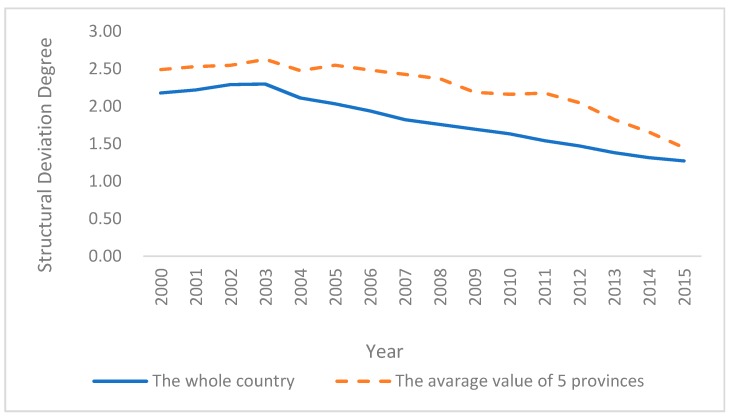
The degree of structure deviation.

We examined the effects of environmental regulation on employment in resource-based provinces in China for the following reasons: on one hand, the proportion of non-renewable resource extraction industries is too high in resource-based areas, and the industrial structure is single. The sustainable development of the economy requires a high-quality living environment and a stable social environment to attract investment. It is urgent to promote the coordinated development of ecology and employment. On the other hand, resource-based areas are the important carrier of the modern industry in China, and the rate of the unemployed is relatively high. The contradiction between industrial pollution control and employment promotion is more prominent. We attempt to resolve the following questions. Can environmental regulation affect employment both directly and indirectly in resource-based areas in China? Are the effects positive or negative? How can we deal with these effects? Focusing on these questions, this paper uses the panel data of five Chinese typical resource-based provinces (Shanxi, Heilongjiang, Jilin, Liaoning, and Inner Mongolia) during 2000–2015 to examine the impacts of environmental regulation on employment. We used the degree of structure deviation to measure the situation of the employment structure. Figure 1 shows that the average value of the five provinces is higher than the value of the whole country. It means that the relationship between the employment structure and the industrial structure is more disequilibrium in these areas. Therefore, we chose the five provinces as the objectives of our study. Finally, we give several policy implications for the coordinated development of the economy and employment based on the empirical results.

## 2. Model and Variables

### 2.1. Hypotheses

On the basis of the existing literature, the direct impact of environmental regulation on employment can be divided into the scale effect and the substitution effect, but the sum is uncertain. Many scholars believe that environmental regulation could reduce employment. The standard explanation for this phenomenon is that such regulation increases production costs, which would raise prices and reduce demand for output, thus reducing employment (at least in a competitive market). Stricter regulation may encourage plants to adopt more efficient production technologies that are capital-intensive and thus, reduce employment. Although this effect seems obvious, a careful microeconomic analysis shows that it is not guaranteed. Even if environmental regulation reduces output in the regulated industry, abating pollution could require additional labor. For example, workers employed in the pollution control sector, such as the labor hired to perform required end-of-pipeline abatement activity. The existing literature does not support the claim that environmental regulation has large negative impacts. For resource-based areas in China, the secondary industry is the mainstay industry and absorbs most of the labor force, while the third industry development is relatively backward. The environmental regulation would hinder the development of the secondary industry, but the government would not allow too many industry enterprises to shut down in a short-term. Owing to the sustainable development of the economy, the negative impact would not be serious. Though regulation may lead to job loss, jobs will also be created. Thus, the following hypothesis is proposed:

**Hypothesis** **1.** Environmental regulation can directly affect employment level in resource-based areas, and the effect is positive.

At the same time, environmental regulation restricts the exploitation of mineral resources and imposes many constraints on the production behavior of enterprises. These polices will inevitably increase the cost of production, leading to the input price of production rising and the scale of industrial investment reducing. While the impact of environmental regulation on the service industry is gentle, this is conducive to the development of the third industry and the upgrade of the industrial structure. The third industry has a leading role in absorbing labor. It is also possible for pollution abatement technologies to be labor enhancing. Besides, Porter’s hypothesis suggests this effect from the strict environmental regulation limit on the emission of waste gas and water in the production process. Confronted with the increased cost of pollution, enterprises must improve the production process and enhance the ability to control pollution by technological innovation to reduce the cost of environmental regulation. Meanwhile, the government will launch the Green Subsidy policy to provide financial support. The technological innovation will lead to changes in labor demand. Hence, environmental regulation would impact employment by the promotion of industrial upgrade and technological progress. Thus, the following hypothesis is proposed.

**Hypothesis** **2.** *Environmental regulation can indirectly affect employment level in resource-based areas through mediating effect of the industrial structure and technological innovation.*

### 2.2. Theoretical Model of Mediating Effect

Based on the above analysis, the mediating effect model is adapted to examine the direct effect and indirect effect of environmental regulation on employment. In consideration of the effect of the independent variable *X* on the dependent variable *Y*, if *X* can affect *Y* through another variable *M*, then *M* is a mediating variable. We use three equations to establish the mediating effect model, as shown in the Equations (1)–(3). The coefficient *c* in Equation (1) denotes the total effect of the independent variable *X* on the dependent variable *Y*. The coefficient *a* in Equation (2) denotes the effect of the independent variable *X* on the mediator variable *M*. The coefficient *b* in Equation (3) denotes the effect of the mediating variable *M* on the dependent variable *Y*. The coefficient *c’* is the direct effect of the independent variable *X* on the dependent variable *Y* after controlling the influence of the mediating variable *M*, *e*_1_~*e*_3_, are the regression residuals.

*Y* = *cX* + *e*_1_ (1)

*M* = *aX* + *e*_2_ (2)

*Y* = *c’X* + *bM* + *e*_3_ (3)

The mediating effect is equal to the indirect effect, which is equal to the product of *a* and *b*. The relationship among *c*, *c’*, *a*, and *b* has the following form:

*c* = *c’* + *ab* (4)

The causal steps approach is the most popular method of testing a mediating effect [15]. First, test the coefficient *c* in Equation (1). Second, test the coefficient *a* in Equation (2) and the coefficient *b* in Equation (3). If the coefficient *c* is significant, and coefficients *a* and *b* are significant too, the mediating effect is significant. Then test the coefficient *c’* in Equation (3). If coefficient *c’* is not significant, the *M* has the role of complete intermediary, otherwise the mediating effect is partial. This method is called the test of joint significance [16].

### 2.3. Empirical Model

According to the analysis, based on Wen juan Yan’s work [17], we set up the following mediating effect model, as shown in the Equations (5)–(7):

(5) $$lnEM_{it}=a_{0}+a_{1}lnEM_{it-1}+a_{2}lnEN_{it}+a_{3}lnGDP_{it}+a_{4}lnHC_{it}+a_{5}lnFDI_{it}+e_{1it}$$

(6) $$lnIS_{it}=b_{0}+b_{1}lnIS_{it-1}+b_{2}lnEN_{it}+b_{3}lnGDP_{it}+b_{4}lnHC_{it}+b_{5}lnFDI_{it}+e_{2it}$$

(7) $$lnEM_{it}=c_{0}+c_{1}lnEM_{it-1}+c_{2}lnEN_{it}+c_{3}lnIS_{it}+c_{4}lnGDP_{it}+c_{5}lnHC_{it}+c_{6}lnFDI_{it}+e_{3it}$$

where, *i* represents the region and *t* represents the period (year). *EM_it_* denotes the level of employment in the current, *EM_it_*_−1_ denotes a variable lagged one period of the employment level, *EN_it_* denotes the stringency of environmental regulation, and *GDP_it_* denotes the gross regional domestic product. *HC_it_* denotes the level of human capital, *IS_it_* denotes industrial structure, *FDI_it_* denotes the foreign direct investment, and *e*_1*it*_~*e*_3*it*_ are the regression residuals.

According to the test of joint significance, if the coefficients of environmental regulation in the three models are all significant, then the partial mediating effect is proved. If the coefficients on environmental regulation in Equations (5) and (6) are significant and the coefficient in Equation (7) is not significant, the complete mediating effect is proved. The coefficient $a_{2}$ measures the total effect of environmental regulation on employment, the coefficient $b_{2}$ measures the impact of environmental regulation on the industrial structure, the coefficient $c_{2}$ measures the direct effect of environmental regulation on employment, and the product of $c_{3}$ and $b_{3}$ measures the indirect effect of environmental regulation on employment, based on changing the industrial structure.

Considering the rigidity of employment, since the employment rate in the current period may be affected by the rate of the previous period, we used lagged differences in the employment in the model by making it dynamic, so we can measure the dynamic effect by Equations (5) and (7). We added the lagged variable of *IS* into Equation (6) for the same reason. In consideration of the correlation between independent variables and error term, the regression analysis should not be directly carried out. Otherwise, the result will be biased. The common way to the problem of auto-correlation and endogeneity in the process of panel regression is to introduce instrumental variables into the equation. We used the lagged variables of the weak exogenous variables as instrumental variables. The first-order difference of the weak exogenous variable is usually used to eliminate the individual effects of the variables and get the consistency estimation results. However, it is difficult to eliminate the autocorrelation between the explanatory variable and the residual variables completely based on the first-order difference. The GMM (Generalized Method of Moments) model can effectively overcome the biased regression results caused by the autocorrelation in the regression.

Similarly, we established the mediating effect model which is based on the variable of technological innovation. The first equation is the same as Equation (5), and the last two equations as follows:

(8) $$lnTECH_{it}=b_{0}\prime +b_{1}\prime lnTECH_{it-1}+b_{2}\prime lnEN_{it}+b_{3}\prime lnGDP_{it}+b_{4}\prime lnHC_{it}+b_{5}\prime lnFDI_{it}+e_{4it}$$

(9) $$lnEM_{it}=c_{0}\prime +c_{1}\prime lnEM_{it-1}+c_{2}\prime lnEN_{it}+c_{3}\prime lnTECH_{it}+c_{4}\prime lnGDP_{it}+c_{5}\prime lnHC_{it}+c_{6}\prime lnFDI_{it}\;+\;e_{5it}$$

where $TECH_{it}$ denotes technological innovation, *e*_4*it*_~*e*_5*it*_ are the regression residuals.

### 2.4. Variables and Data

#### 2.4.1. The Dependent Variable

The objective of this study is to prove the impact of environmental regulation on employment in resource-based provinces. We used the numbers of workers as a proxy of employment, in consideration of the data availability. We used the data of the industrial enterprises whose profits achieve an annual revenue of 3.3 million dollars or more from their main business operations. We used five typical resource-based provinces’ (Shanxi Province, Heilongjiang Province, Jilin Province, Liaoning Province, and Inner Mongolia) panel data from the “China Labor Statistical Yearbook” for the period 2000–2015.

#### 2.4.2. The Core Independent Variable

The core independent variable is the environmental regulation, because there are no data in our database about environmental regulation. Many scholars use per capita as a proxy of environmental regulation, that is, as the level of income rising, environmental regulation is more stringent [18]. Domazlicky used the emission of different contaminants as a measure of a country’s environmental regulation [19]. Less pollution emission means that more stringent environmental regulation has been enforced. Many scholars in China use the investment of pollution control as the index of environmental regulation. We argue that the investment of individual pollution control cannot reflect the intensity of environmental regulation in different regions accurately. Hence, this research uses the ratio of investment in industrial pollution control to industrial output as a proxy indicator. The data is from the “China Environmental Statistical Yearbook.”

#### 2.4.3. Mediator Variables

The first mediator variable is industrial structure. The general way is to use the ratio of the output in three industries to GDP as a proxy variable. Considering that environment-intensive industries are concentrated in the secondary industry, we used the ratio of total output of the third industry to the total output of the secondary industry. The data comes from the “China Statistical Yearbook”.

The second mediator variable is technical innovation. There are generally three indicators for technical innovation: R & D expenditures [20,21], the number of patent applications [22,23,24], and sales derived from new products [25,26]. Considering the availability of data and the research objective, we adopted the number of patent applications as a proxy for technical innovation. The data is from the “China Science and Technology Statistical Yearbook”.

#### 2.4.4. Control Variables

The first control variable is the level of economic development (GDP), in the 20th century. The American economist Okun argued that if the GDP increases by 2%, the unemployment rate will fall by about 1%. The relationship may not be accurate, but the economic growth and the employment rate must be inseparable. According to Engel’s law, the structure of consumption will be improved with the development of the economy, and the change of consumption structure will bring a positive impact to the industrial structure and to technological innovation. Thus, it must be controlled in the model. To eliminate the impact of inflation, we used a GDP deflator to transform nominal GDP into a constant-price GDP of 2000. 

The second control variable is human capital (HC), considering that the impact of human capital on employment should not be ignored. We used the average education years of workers as a proxy. The data is calculated as follows. According to China’s education system, the education level is divided into five groups, namely college and above, high school, junior high school, primary school, and illiterate semi-illiterate. The weight of various types of personnel is set, based on the number of years of education. Specifically, we assign 1 to illiterate and semi-illiterate people, 6 to people with primary school education, 9 to people with junior high school education, 12 to people with high school education, and 16 to people with college and above education. Considering the availability of data, we put graduate, undergraduate, and college specialists into a group, and ignored the differences between college, undergraduate, master, and doctoral education. Finally, the per capita education period is obtained by a weighted summation, expressed by the equation:

$$\begin{array}{c} \mathrm{per}\;\mathrm{capita}\;\mathrm{years}\;\mathrm{of}\;\mathrm{education}\\ =\frac{\sum \mathrm{the}\;\mathrm{number}\;\mathrm{of}\;\mathrm{people}\;\mathrm{receiving}\;\mathrm{different}\;\mathrm{levels}\;\mathrm{of}\;\mathrm{education}\times \mathrm{weight}}{\mathrm{the}\;\mathrm{sum}\;\mathrm{of}\;\mathrm{the}\;\mathrm{number}\;\mathrm{of}\;\mathrm{different}\;\mathrm{levels}\;\mathrm{of}\;\mathrm{education}} \end{array}$$

The data is from the “China Labor Yearbook” and the “China Statistical Yearbook”.

The third control variable is foreign direct investment (FDI). Foreign direct investment can promote technical progress through the competition effect, the spillover effect, and the correlation effect, which are all conducive to energy conservation and emissions reduction [27]. We used the ratio of the actual foreign investment to GDP to measure foreign direct investment.

All data were logarithmically processed. It is often adopted to reduce the fluctuation of the data, eliminate the heteroscedasticity of the time series, and transform the nonlinear relation into a linear relationship. At the same time, it can reduce the extreme value, any non-normal distributions, and heteroscedasticity of the variables.

## 3. Results

First of all, we processed the data through a stationary test before the econometric regression analysis. This paper conducts the unit root test in three ways, that is, Levin, Lin, and Chu (LLC), ADF-Fisher, and PP-Fisher. The results show that all the variables are stationary sequences (See Table 1). Prior to estimating the parameters of the panel data, it is necessary to examine the cointegration relations between employment, environmental regulation, industry structure, and technical innovation to avoid spurious regression. Pedroni panel cointegration was carried out and the results indicate that panel cointegration relationships exist between employment and environmental regulation, environmental regulation and industry structure, and environmental regulation and technical innovation (See Table 2). Furthermore, variance inflation factors (VIFs) are less than 10, indicating that there is no evident multicollinearity between variables. Therefore, we can use the above econometric models to perform the regressions.

Considering that the equations contain the lagged dependent variables, the fixed effects model cannot deal with the endogenous explanatory variables. There are two main methods to estimate ordinary dynamic panel models: difference GMM and system GMM. Since the system GMM regression estimation can deal with less finite sample bias problems and can better increase accuracy, it has been widely used in estimating ordinary dynamic panel models. We chose the system GMM regression method to estimate ordinary dynamic panel models. The descriptive statistic results of variables in the econometric regression model are shown as Table 3.

The result of AR (1) shows that there is first-order autocorrelation between variables, and the result of AR (2) shows that there is no second-order serial correlation between variables. According to the Arellano–Bond auto-correlation (AR) test, we find that the model based on system GMM estimator is valid. The result of the Sargan test shows that the instrumental variables are not over-identified.

## 4. Discussion

According to the results in the Table 3, we examined the mediating effect following the causal steps approach. Firstly, the coefficient of environmental regulation in Equation (5) is significantly negative at the 0.01 significance level. The test of joint significance indicated that the mediating effect model can be established. Secondly, the coefficient of environmental regulation in Equation (6) is significantly positive at the 0.01 significance level, and the coefficient of industrial structure in Equation (7) is significantly negative at the 0.01 significance level. Thus, the indirect effect is proved. Thirdly, the coefficient of environmental regulation in Equation (7) is significantly negative at the 0.01 significance level, which means that the model has both direct and indirect effects. The coefficient of environmental regulation in Equation (7) is significant, so the partial mediating effect exists. The mediating effect is 0.0099, and the total effect is 0.03. In conclusion, the industrial structure plays a mediating role between environmental regulation and employment in these resourced-based provinces of China. The mediating effect accounts for 35% of the total effect of environmental regulation on employment. From the estimation results of Equation (5), the coefficient on environmental regulation is 0.028, which shows that the impact of the environmental regulation on employment in China’s resource-based provinces is positive. This confirms Hypothesis 1. In Equation (6), we found a significant effect of 0.041 for the upgrade of the industrial structure in China’s resource-intensive provinces. The coefficient of environmental regulation in Equation (7) apparently suggests that environmental regulation has a positive impact on employment. The mediating effect means that an increase in regulatory stringency leads to the increase of employment via industrial upgrades.

Similarly, Table 3 indicates the mediating effects of technological innovation. The coefficients of environmental regulation in Equations (5) and (8), and the coefficient of technological innovation in Equation (9) are all significantly negative at the 0.01 significance level. The mediating effect is −0.0063, and the total effect is 0.028. The coefficient of environmental regulation in Equation (9) shows that there is a significantly positive effect on technological innovation, which is consistent with the “weak” Potter hypothesis. Appropriate environmental regulation could promote the technological innovation. However, the impact is slight because technological progress is a long-term process. Besides, the impact of environmental regulation on technological progress has not yet developed completely. The mediating effect is negative, indicating that environmental regulation cannot promote employment by activating technological innovation. Technological innovation is accompanied by the usage of machinery and equipment, which will lead to the substitution effect of machinery and equipment on the labor force. This will cause a reduction of the number of employees. The technological innovation in the pollution control sectors occupies a small proportion. The Hypothesis 2 is verified. Environmental regulation can affect employment by promoting industrial upgrading and technological innovation. The mediating effect of the industrial upgrading is positive while the mediating effect of the technological innovation is negative.

As for control variables, the lag coefficient of employment is significantly positive at the 0.01 significance level. It reveals that the employment scale of the previous period will have an obviously positive effect on the current period and the dynamic panel data model is appropriate. The coefficients of GDP are significantly positive at the 0.01 significance level, which means that economic development is conducive to employment. The coefficients of human capital are significantly negative at the 0.01 significance level. With the improvement of education, enterprises tend to employ workers with a good educational background and with good knowledge. The coefficients of foreign direct investment are not significant, indicating a lack of importance of FDI in promoting employment in these resource-based areas.

## 5. Conclusions

This study analyzes the mechanisms of employment effects based on environmental regulation, and empirically examines these effects. We established two mediating effect models based separately on industrial upgrading and technological innovation. The dynamic econometric results show that environmental regulation has direct positive impacts on employment, and has positively mediated the effects on employment by inducing industrial upgrades. Besides, the mediating effects of technological innovation are negative.

According to the result of our empirical analysis, there is no conflict between environmental regulation policy and employment. Our research has some policy implications on the coordination of the development of employment and the environment. In recent years, the economy grew rapidly in resource-based areas of China, but the existence of “extensive” growth has engendered severe population-, resource- and environment-related problems. The best way is to establish a new diversified leading industry system instead of the single heavy industrial structure. The first step is to strengthen environmental regulation and pollution control. If the environmental regulation is weak, enterprises can still expand their production scale with a low cost of input. In this situation, some of the enterprises may choose to reduce employees to save costs, facing the extra cost of pollution control, rather than take actions to reduce pollution. Strict environmental regulation can force upgrades of the industrial structure, absorbing a great deal of the labor force and saving environmental resources. We can learn from the effect of the federal Clean Air Act in the U.S., that the stricter regulation has not lead to a reduction in employment. Furthermore, there is some evidence that the regulations have increased productivity and job creation. Besides, the government should stimulate the manufacturing industry to give priority to the innovation of energy-conserving technologies by way of fiscal subsidies, tax preference, etc., and should conduct technical innovation relating to green production technologies. At the same time, the government ought to encourage promotion and application of energy-conserving and environmental-protection products through government purchases, subsidies for low-carbon products and the market share of green products. These policies are both to the benefit of reducing pollution and promoting the upgrade of the industrial structure. Lastly, policymakers should prepare a variety of educational programs, to educate workforces on how to be able to fit into new jobs generated by technological changes and to help them learn more advanced skills. It can enable them to keep their competence by quickly adjusting to the rapid technological changes. Consequently, the rate of unemployment caused by technological innovation will decline. This research can also be applied to other resource-based countries, such as Russia, Brazil, and Australia. In these countries, the workers in the oil, coal, and natural gas industries are facing job losses due to the rise of energy prices. These polices above may help to reduce unemployment.

## Figures and Tables

**Table 1:** The results of the stationary test.

Varible	LLC Test	ADF-Fisher Test	PP-Fisher Test	Results
***lnEM***	−14.452 ***	117.363 ***	145.667 ***	Stationary
***lnEN***	−9.624 ***	93.469 ***	134.234 ***	Stationary
***lnIS***	−11.236 ***	112.344 *	157.342 ***	Stationary
***lnTECH***	−13.244 ***	115.379 ***	146.383 ***	Stationary
***lnHC***	−12.981 ***	103.451 *	136.924 *	Stationary
***lnGDP***	−9.343 ***	87.364 ***	127.474 ***	Stationary
***lnFDI***	−7.435 ***	78.451 ***	131.712 ***	Stationary

Note: * significant at 0.1 level; *** significant at 0.01 level.

**Table 2:** The results of the cointegration test.

Statistics	Test Results			
	Test Methods	*EM* and *EN*	*EN* and *IS*	*EN* and *TECH*
**Intragroup statistics**	**Panelv-Stat.**	9.353 ***	2.319 **	3.271 **
	**Panelpp-Stat.**	−0.392	0.549	1.934
	**Panelr-Stat.**	−8.258 ***	−5.391 ***	−0.817
	**PanelADF-Stat.**	−9.387 ***	−2.345 **	−2.374 **
**Intergroup statistics**	**Group-Stat.**	1.832	3.275 **	3.571 **
	**GroupPP-Stat.**	−11.639 ***	−6.438 ***	−6.658 ***
	**GroupADF-Stat.**	−10.375 ***	−3.234 **	−7.473 ***

Note: ** significant at 0.05 level; *** significant at 0.01 level.

**Table 3:** Estimation results of the econometric regression model.

Type	Variables	Equation (5)	Equation (6)	Equation (7)	Equation (8)	Equation (9)
**Dependent variable**	***lnEN***	0.028 *** (11.482)	0.041 ** (2.358)	0.047 *** (11.096)	−0.036 *** (2.569)	0.021 *** (11.082)
**Control variables**	***lnGDP***	0.453 *** (−3.764)	0.043 *** (2.734)	1.329 *** (−4.331)	0.133 * (1.814)	0.540 * (−1.754)
	***lnHC***	−1.324 *** (−5.847)	0.051 ** (−2.172)	−1.109 *** (−5.622)	0.643 *** (2.786)	−1.673 *** (−4.919)
	***lnFDI***	0.405 (1.643)	0.115 *** (3.245)	−0.276 (1.252)	−0.026 (−0.396)	0.383 (1.425)
**Lag variables**	***lnEM (−1)***	0.114 ** (2.014)		0.041 *** (3.545)		
	***lnIS (−1)***		0.907 *** (7.735)			
	***lnTECH (−1)***				0.738 *** (8.525)	
**Mediator variable** **s**	***lnIS***			0.243 *** (5.115)		
	***lnTECH***					0.174 *** (2.432)
**Cons**		−0.813 (−0.346)	−1.634 (−4.135)	8.579 (3.191)	−7.774 (−2.633)	3.835 (0.934)
**Sargan test**		20.332 [0.973]	21.235 [0.740]	23.892 [0.693]	25.034 [0.713]	24.677 [0.841]
**AR (1)**		−4.051 [0.039]	−4.019 [0.047]	−3.076 [0.035]	−2.981 [0.053]	−3.457 [0.017]
**AR (2)**		−0.674 [0.491]	−0.756 [0.937]	−0.438 [0.539]	−0.573 [0.613]	−0.497 [0.721]

Note: *, **, *** means statistically significant at the 1%, 5% and 10% levels respectively. The data in parentheses are *Z*-value. The numbers in the square brackets are *p*-value. The data of AR (1) and AR (2) represent respectively the residuals of first-order difference and second-order difference by the Arellano–Bond auto-correlation test.
